# Validity of dried blood spot testing for sexually transmitted and blood-borne infections: A narrative systematic review

**DOI:** 10.1371/journal.pgph.0003320

**Published:** 2024-06-14

**Authors:** François Cholette, Simone Périnet, Bronwyn Neufeld, Maggie Bryson, Jennifer Macri, Kathryn M. Sibley, John Kim, S. Michelle Driedger, Marissa L. Becker, Paul Sandstrom, Adrienne F. A. Meyers, Dana Paquette

**Affiliations:** 1 National Sexually Transmitted and Blood Borne Infection Laboratory, National Microbiology Laboratory at the J. C. Wilt Infectious Diseases Research Centre, Public Health Agency of Canada, Winnipeg, Canada; 2 Department of Medical Microbiology and Infectious Diseases, University of Manitoba, Winnipeg, Canada; 3 Sexually Transmitted and Blood Borne Infection Surveillance Division, Centre for Communicable Diseases and Infection Control, Public Health Agency of Canada, Ottawa, Canada; 4 Horizontal Surveillance Operations Division, Centre for Corporate Surveillance Coordination, Public Health Agency of Canada, Ottawa, Canada; 5 Public Health Data Science and Systems, Data Management, Innovation and Analytics, Public Health Agency of Canada, Ottawa, Canada; 6 Department of Community Health Sciences, University of Manitoba, Winnipeg, Canada; 7 George & Fay Yee Centre for Healthcare Innovation, University of Manitoba, Winnipeg, Canada; 8 Office of Population and Public Health, Indigenous Services Canada, Ottawa, Canada; Eduardo Mondlane University: Universidade Eduardo Mondlane, MOZAMBIQUE

## Abstract

Testing for human immunodeficiency virus (HIV) and hepatitis C virus (HCV) using dried blood spot (DBS) specimens has been an integral part of bio-behavioural surveillance in Canada for almost two decades, though less is known regarding the use of DBS in surveillance of other sexually transmitted and blood-borne infections (STBBI). A systematic review was conducted using a peer-reviewed search strategy to assess the current evidence regarding the validity of STBBI testing using DBS specimens. Eligibility criteria included studies reporting use of DBS specimens for STBBI testing with either commercially available or “in-house” tests in populations 15 years of age or older. Studies reporting a measure of validity such as sensitivity, specificity, positive and negative predictive values were eligible for inclusion. Quality of studies and risk of bias were assessed using the QUADAS-2 tool. A total of 7,132 records were identified. Of these, 174 met the criteria for inclusion. Among the studies that reported validity measures, a substantial proportion demonstrated high sensitivity (≥90%) in 62.5% of cases (*N* = 334/534 sensitivity measurements), and high specificity (≥90%) was observed in 84.9% of instances (*N* = 383/451 specificity measurements). However, the quality of the studies varied greatly. Our findings support the validity of the use of DBS specimens in STBBI testing where sufficient evidence was available, but validity is highly dependent on thorough method development and validation.

## Introduction

Dried blood spot (DBS) specimens consist of blood spotted and dried on filter paper. They have been used for biological sampling in clinical and research settings for nearly a century [1, 2]. Compared to conventional biological specimens like plasma or serum, DBS offers several advantages, including high acceptability among research participants [3] and compatibility with self-collection [4]. DBS specimens are also stable at room temperature and do not require refrigeration during transportation, providing opportunities for biological sampling in remote and isolated communities [5]. DBS sampling is typically deployed in resource-constrained settings for infectious disease diagnostics [6], surveillance [7], and patient monitoring [8], but it also has the potential to be an effective tool for infectious disease surveillance in high resource settings like Canada given the country’s numerous northern, remote, and isolated communities scattered across challenging geography. DBS sampling has also been shown to have a high acceptability as an approach of screening for sexually transmitted and blood-borne infections (STBBIs) in communities across Canada [3, 9].

DBS sampling was thrust into the spotlight during the COVID-19 pandemic, as it became routine for surveillance and epidemiological studies while limiting face-to-face interactions and overcoming healthcare staff shortages by allowing individuals to reliably collect their samples at home [10]. For example, Statistics Canada’s Canadian COVID-19 Antibody and Health Survey (CCAHS) relied on DBS self-collection to assess COVID-19 seroprevalence nationally (www23.statcan.gc.ca/imdb/p2SV.pl?Function=getSurvey&SDDS=5339). As a result, lessons learned from implementing large-scale DBS sampling for SARS-CoV-2 surveillance could inform STBBI surveillance moving forward. In many high resource settings, the prevalence of STBBIs such as HIV, HCV and syphilis remains stable or is increasing despite the presence of effective public health interventions [11, 12]. While case-based surveillance provides information on those who are tested through routine health care, sentinel surveillance can provide insights into the true burden of STBBIs, especially among vulnerable populations who either chose not to engage with the health care system or have limited access to health care facilities.

Diagnostic assays have shown excellent clinical performance on DBS specimens. Most validation studies have been performed for HIV screening, but more recent studies have demonstrated the feasibility of using DBS specimens for the diagnosis of viral hepatitis [13]. Additionally, DBS have been widely used in quantitative assays for HIV viral load monitoring [14]. However, it is difficult to ascertain if DBS are a suitable specimen for STBBI surveillance considering the many experimental conditions (i.e. DBS punching and elution protocols), combinations of reference and index tests, and study populations. Special consideration is also needed for key populations where dual-infections are more prevalent, including HCV/HIV co-infection among people who inject drugs and HIV/syphilis co-infection in men who have sex with men [15, 16]. The presence of dual-infections is important to consider as it may have the potential to impact assay performance [17]. However, most validation studies focus on a single STBBI and therefore performance metrics are presented for a single index test, pathogen, and population, making it difficult to draw broader conclusions on the validity of using DBS specimens for STBBI surveillance.

The objective of this systematic review is to compile data on measures of validity to ascertain if the current literature supports the use of DBS for the detection of the following STBBI: HIV-1, HIV-2, hepatitis viruses (A, B, and C), herpes simplex virus (type 1 and 2), human T-cell lymphotropic virus (type 1 and 2), human papilloma virus, chlamydia (*Chlamydia trachomatis*), gonorrhea (*Neisseria gonorrhoeae*), and syphilis (*Treponema pallidum*). Measures of validity include sensitivity, specificity, positive predictive values (PPV), negative predictive values (NPV), limit of quantification (LOQ), and/or limit of detection (LOD). Additionally, we aim to identify factors which may influence test performance.

## Methods

### Information sources

Peer-reviewed original research was identified by searching Excerpta Medica dataBASE (EMBASE), Medical Literature Analysis and Retrieval System Online (Ovid MEDLINE) and Elsevier Scopus. Grey literature was identified by searching key websites including Public Health Ontario, BC Centre for Excellence in HIV/AIDS, Canadian AIDS Treatment Information Exchange (CATIE), Institut national d’excellence en santé et services sociaux, Open Grey, Public Health Agency of Canada, American Society of Microbiology, Infectious Disease Society of America, American Society of Virology, International AIDS Society, International Society for Sexually Transmitted Diseases Research, Conference on Retroviruses and Opportunistic Infections (CROI), International AIDS Conference, Centre for Disease Control (CDC), Public Health England, European Centre for Disease Prevention and Control, World Health Organization (WHO) and the French National Agency for AIDS Research (ANRS).

### Search strategy

Literature searching was conducted under the guidance of a librarian (Janice Linton, University of Manitoba) using a peer-reviewed search strategy [18]. The search strategy consisted of both controlled vocabulary such as the National Library of Medicine’s MeSH (medical subject heading) and keywords (S1 Text). Retrieval was limited to human populations, English and French language documents, and results were not limited by publication date up to August 2023. We did not register or publish a protocol for this review.

### Eligibility criteria

Populations were eligible if they provided DBS specimens tested for the following STBBIs: human immunodeficiency virus type 1 and 2 (HIV-1 and HIV-2), hepatitis virus A, B, and C (HAV, HBV, and HCV), human T-cell lymphotropic virus type 1 and 2 (HTLV-1 and HTLV-2), human papilloma virus (HPV), *Chlamydia trachomatis* (chlamydia), *Neisseria gonorrhea* (gonorrhea) and *Treponema pallidum* (syphilis). Studies were eligible if they were conducted on populations 15 years of age or older, regardless of socio-demographic characteristics and setting.

The intervention of interest was either commercially available or in-house tests used to detect STBBIs from DBS specimens. Inclusion criteria for commercial tests were limited to 3^rd^ generation or greater enzyme immunoassays (EIAs) and nucleic acid tests. Older HIV testing methodologies (i.e., 1^st^ and 2^nd^ generation EIAs) are likely no longer manufactured [19], and were therefore excluded from this review.

The intervention was compared to either commercially available or in-house tests used to detect STBBIs from “gold-standard” biological specimens (ex: whole blood, plasma and serum) used for routine STBBI testing.

Included studies had to report measures of the intervention’s validity such as sensitivity, specificity, positive predictive values (PPV), negative predictive values (NPV), limit of quantification (LOQ) and/or limit of detection (LOD).

### Exclusion criteria

Peer-reviewed or grey literature were excluded if (1) the pathogen of interest was not included in the list of STBBIs mentioned above, (2) measures of the testing method’s validity were not reported, (3) biological specimens other than blood were collected on filter paper, (4) DBS were used to measure adherence to pre-exposure prophylaxis (PrEP) or anti-retroviral therapy (ART) by quantifying drug concentrations, (5) DBS were analyzed for the purpose of investigation of drug resistance or genotyping, (6) participants were under 15 years of age, (7) the intervention was out of scope (i.e., blood dried on matrix other than filter paper), (8) the work was not original research, or (9) the reports were in a language other than English or French.

### Document screening

Titles and abstracts were initially screened by one reviewer (FC), while a second reviewer (SP and BN) subsequently peer-reviewed 10% of these initial screened documents. Following this preliminary screening, a full-text review of selected articles was performed by the same initial reviewer (FC) to determine their eligibility for inclusion. Similarly, 10% of these full-text reviews was subjected to peer-review be a second reviewer (SP and BN). In cases of disagreement regarding the inclusion of specific articles, a third reviewer (MB) was consulted to make the final decision.

### Data extraction

Data were collected from each document on the inclusion list (e.g., study population, sampling method, sample size, index test, reference test, STBBI, DBS preparation method, sensitivity, specificity, accuracy, PPV, NPV, LOQ and/or LOD) and entered into a standardized table. For each document, descriptive data were also extracted, including information on the authors, year of publication, country, setting, participant characteristics, description of the intervention, description of the comparators, and other key findings related to the research question. One reviewer (FC) extracted descriptive and outcome data, while a second reviewer (SP and BN) was responsible for verifying the data extraction for accuracy.

### Quality assessment

The QUADAS-2 tool was used to assess the quality and risk of bias of each document retained for data extraction to critically appraise the validity of DBS testing [20]. One reviewer (FC) assessed the quality of each document using the QUADAS-2 tool available from the QUADAS website (www.quadas.org), and a second reviewer (SP and BN) verified the assessment. A third reviewer, MB, resolved any disagreements.

### Data analysis

Data extracted from selected documents were synthesized through a narrative synthesis approach [21]. A narrative approach to synthesis was chosen because we had anticipated significant heterogeneity among documents in terms of context, patient populations, and index tests. Synthesized findings were compiled in tabulated form organized by STBBI, population, sample size, index test, and collection method. In addition, inductive thematic analysis was conducted in order to identify key themes and relationships in included studies.

## Results

**Fig 1:**
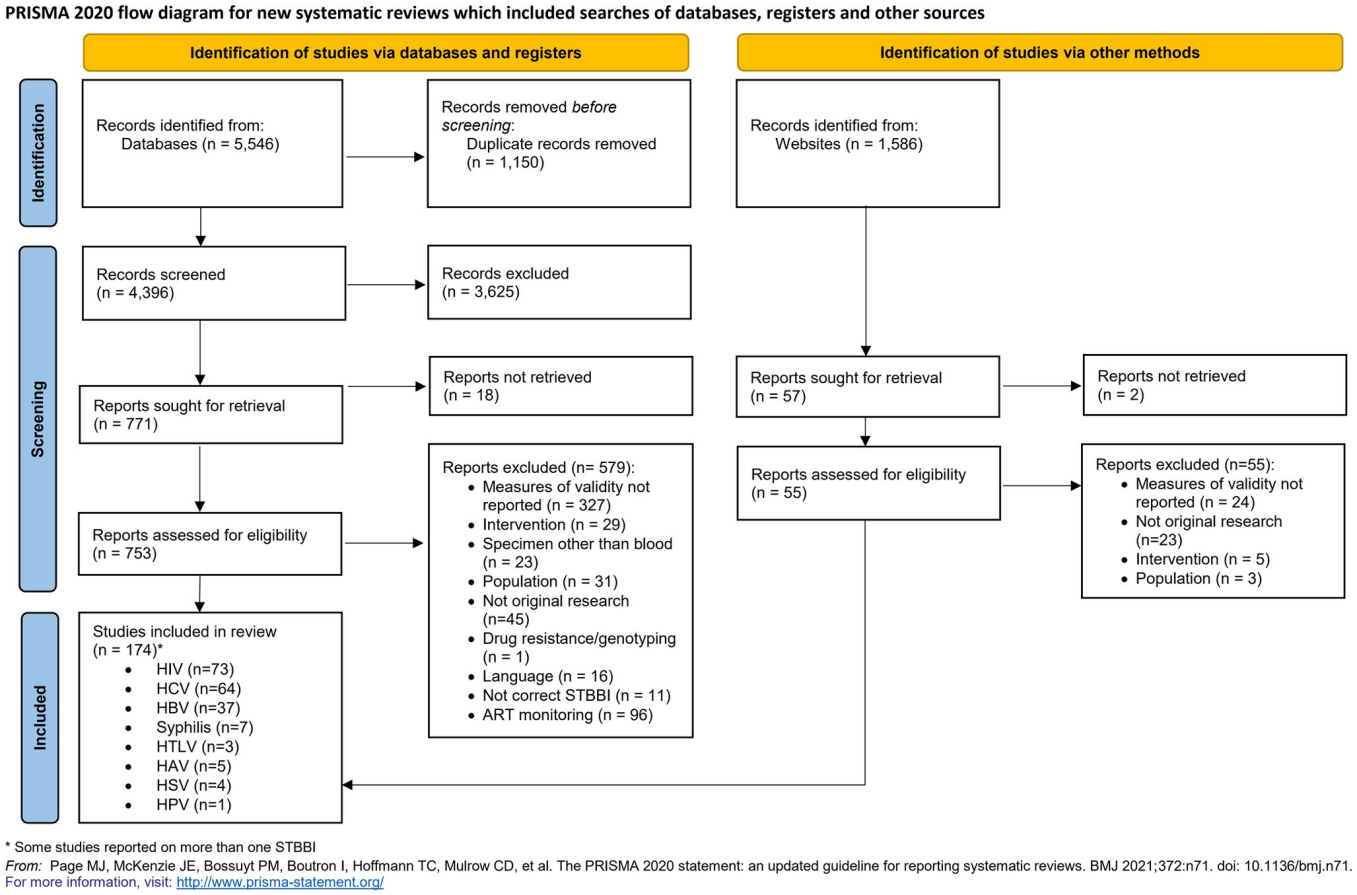
PRISMA flow diagram.

We identified 5,546 abstracts in database searches and 1,586 reports from grey literature. After 1,150 duplicates were removed, 4,396 titles and abstracts were screened, and 3,625 excluded. 753 reports were assessed for eligibility, with 579 further excluded. A total of 174 studies met the inclusion criteria and were included in the review. A PRISMA flow diagram [22] representing the study selection is shown in Fig 1.

### Study characteristics

Characteristics of included studies are summarized in the S1 Data. A large proportion of included studies reported on the use of DBS for HIV testing (*n* = 73; 42.2%). The remaining studies reported on HCV (*n* = 64; 37.0%), HBV (*n* = 37; 21.4%), syphilis (*n* = 7; 4.0%), HAV (*n* = 5; 2.9%), HSV (*n* = 4; 2.3%), HTLV (*n* = 3; 1.7%), and HPV (*n* = 1; 0.6%). No studies reported on chlamydia or gonorrhea. Several studies reported on more than one STBBI (for example: Villar et al. 2011 [23]), leading to a total of outcomes above the total number of studies included in this systematic review.

### STBBI test performance with DBS specimens

**Fig 2:**
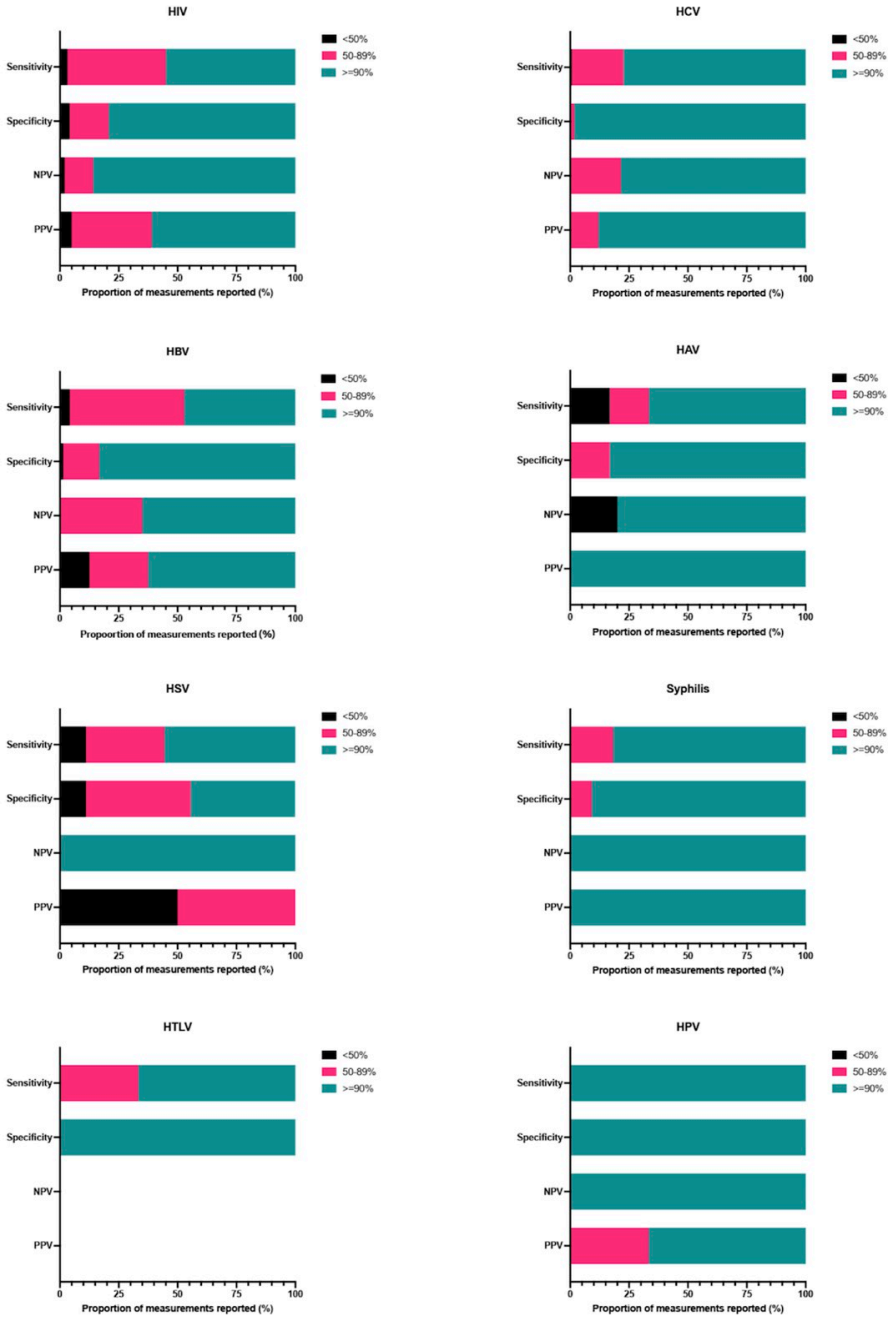
Proportion of reported performance measurements with <50%, 50–89%, or ≥90% sensitivity, specificity, negative predictive values (NPV), and positive predictive values (PPV).

Overall, included studies demonstrated high sensitivity and specificity for STBBI testing with DBS specimens (Fig 2). HIV studies reported a wide range of values for sensitivity (9.0 to 100%) and specificity (4.0 to 100%). The majority of sensitivity measurements (*n* = 137; 55.0%) were equal to or above 90.0%. Specificity measurements were also high, with most observations (*n* = 172; 79.3%) above 90.0%. HCV studies reported sensitivity and specificity ranging from 36.0% to 100% and 85.7% to 100%, respectively. Most sensitivity (*n* = 130; 77.4%) and specificity (*n* = 116; 98.3%) measurements concerning HCV were equal to or above 90.0%. HBV studies reported sensitivity and specificity ranging from 40.0% to 100% and 2.5% to 100%, respectively. Approximately half of sensitivity measurements (*n* = 34; 47.2%) and most specificity (*n* = 60; 83.3%) measurements concerning HBV were equal to or above 90.0%. Studies involving syphilis reported high sensitivity and specificity, with ranges of 90.0% to 100% and 99.0% to 100%, respectively. HAV studies reported sensitivity and specificity ranging from 31.0% to 100% and 75.0% to 100%, respectively. Most sensitivity (*n* = 4; 66.7%) and specificity (*n* = 5; 83.3%) measurements concerning HAV were equal to or above 90.0%. HSV studies reported sensitivity and specificity ranging from 9.0% to 100% and 4.5% to 100%, respectively. Most sensitivity measurements (*n* = 5; 55.6%) and almost half of specificity measurements (*n* = 4; 44.4%) concerning HSV were equal to or above 90.0%. HTLV studies reported a range in sensitivity of 81.0% to 100%, while all included studies reported 100% specificity. The single HPV study reported an overall sensitivity and specificity of 98.0% and 92.0%, respectively. Approximately one-quarter of studies (*n* = 47; 27.8%) reported LOD and/or LOQ values (S1 Table). A meta-analysis was not undertaken due to the significant methodological heterogeneity among the included studies. This led us to identify several parameters that could influence index test performance.

### Test cut-offs

Studies generally reported improved test performance by adjusting cut-off values–typically optimized for serum/plasma by manufacturers–for DBS specimens (S2 Table). For example, García-Cisneros et al. (2019) found that the IgG-G2 Human ELISA test (Human Diagnostics, Germany; HSV-2) performed better on DBS (specificity of 4.5% [95% CI = 3%, 6.5%] versus 87.1% [95% CI = 81.2%, 91.4%]) when using a cut-off value based on a receiver operating characteristic (ROC) curve. Villar et al. (2011) also observed lower sensitivity (95.5% [95% CI = 84.5%, 99.4%] versus 97.6% [95% CI = 87.4%, 99.9%]; ETI-MAK-4 test, DiaSorin) and specificity (81.3% [95% CI = 70.7%, 89.4%] versus 97.3% [95% CI = 90.7%, 99.7%]; ETI-AB-AUK-3 test, DiaSorin) values when relying on manufacturer recommended cut-offs compared to cut-off values based on ROC curve analysis for the detection of HBV in DBS specimens. Ultimately, we observed that the choice of cut-off value (manufacturer recommended versus established in-house) influenced test performance when analyzing DBS specimens.

### DBS specimen preparation

DBS specimens prepared from venous blood instead of capillary blood (i.e., finger pokes) appear to produce higher sensitivity and specificity values (S3 Table). This was particularly evident with regards to HIV. Multiple studies reported better HIV serological and nucleic acid test performance when analyzing DBS specimens prepared from venous blood [24–26]. A few papers examined test performance between DBS specimens prepared from venous and capillary blood for HCV testing, with the majority of those finding similar or greater sensitivity when using DBS specimens prepared with venous blood. Prinsenberg et al. (2020) reported a small advantage in sensitivity (96.4% [95% CI = 81.7%, 99.9%] versus 95.7% [78.1%, 99.9]) when using venous blood to prepare DBS specimens, while Tran et al. 2020 [27] and Vetter et al. 2021 [28] both reported nearly identical test performance with DBS prepared from venous and capillary blood (S3 Table). In general, how DBS are prepared (venous versus capillary blood) could influence test performance, with DBS prepared from venous blood contributing to better test performance (approximately 1% to 5% better sensitivity), though the observed difference does may not be significant due to overlapping confidence intervals in several cases.

### Dual infections

The presence of dual infections may influence test performance depending on the STBBI of interest (S4 Table). This was particularly evident when testing for HBV and HCV on DBS collected from individuals living with HIV. Flores et al. 2017 [29] reported lower sensitivity and specificity in persons living with HIV compared to those without HIV for the detection of both HBV surface antigens (HBsAg) and anti-hepatitis B core total antibodies (Anti-HBc) on the Elecsys platform (Roche; S4 Table). Similar findings have also been observed in HCV testing. De Crignis et al. 2010 [30], Saludes et al. 2018 [31], and Flores et al. 2018 [32] all reported reduced HCV test sensitivity in people living with HIV (S4 Table). In contrast, Flores et al. 2021 [33] reported increased HCV test performance in people living with HIV compared to those who were not, but chronic kidney disease among study participants may have confounded these findings. As heterogeneity between studies was considerable, it is difficult to ascertain the exact impact of dual infections on assay performance with certainty. Nonetheless, the presence of potential co-infections should be taken into consideration when validating a test for use with DBS specimens.

### Antiretroviral therapy

In studies which included people living with HIV, assay performance was typically assessed in patients undergoing antiretroviral therapy (ART) in comparison to ART-naïve patients to detect treatment failure (S5 Table). Balinda et al. 2016 [34] reported differences in the performance of an in-house HIV RT-qPCR assay in participants living with HIV who were undergoing ART compared to those who were ART-naïve (79.4% sensitivity, 54.5% specificity), with the highest performance observed in patients who had undergone ART for longer periods (12–36 months; 88.9% sensitivity, 98.1% specificity). Similarly, Taeib et al. 2018 [35] reported greater specificity in patients on ART for ≥6 months compared to those on ART for <6 months but, found no difference in sensitivity.

### Quality assessment

**Fig 3:**
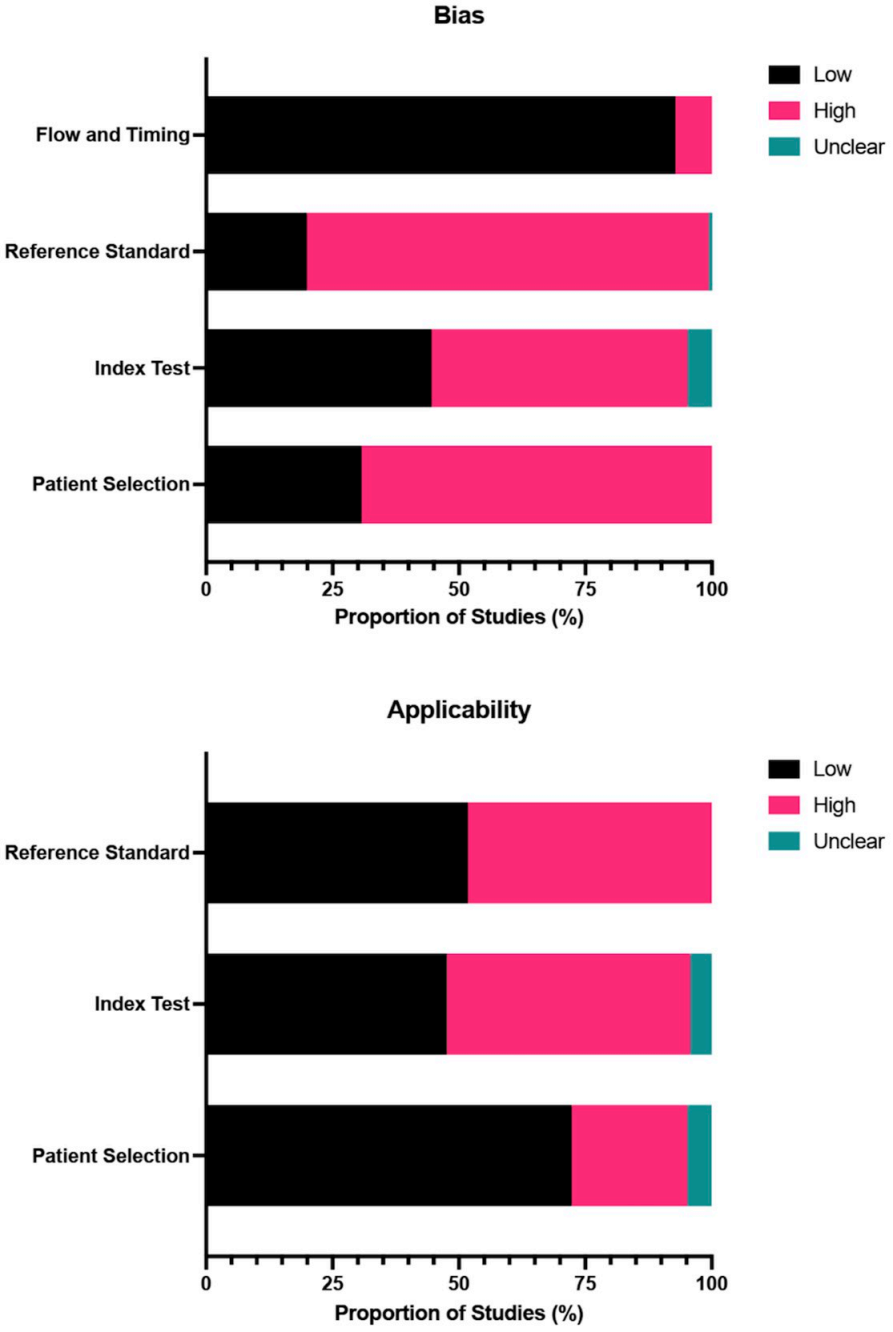
Proportion of studies with low, high, or unclear risk of bias and concerns regarding applicability based on the QUADAS-2 tool.

All 174 included studies were assessed for quality using the QUADAS-2 tool (Fig 3). The overall quality of studies was low, with high scores in risk of bias and applicability. Risk of bias was found to be high across most domains, with 116 studies (67.1%) rated as having high risk of bias in patient selection, 85 (49.1%) as high risk of bias for the index test, and 137 (79.2%) as high risk of bias for the reference standard. In addition, a small number (*n* = 12; 6.9%) of studies were rated as high risk of bias related to flow and timing. Similarly, a significant proportion of studies were rated as high concern regarding the applicability of the study to the research question. The proportion of studies rated as high concern regarding the applicability of population, index test, and reference standard was found to be 23.7%, 46.8%, and 48.6%, respectively.

## Discussion

This review describes the validity of using DBS specimens for STBBI testing. DBS specimens showed high sensitivity compared to the reference standard of plasma or serum in the included studies encompassing a wide range of experimental conditions, demonstrating a promising opportunity for the adoption of DBS specimens in STBBI testing globally. The high sensitivity and specificity observed across studies provide evidence for the suitability of DBS for the surveillance of STBBIs, including those not routinely detected in DBS specimens, such as HAV, HTLV and syphilis. However, it is important to acknowledge that the bulk of these findings, approximately 90%, pertain to HIV, HCV, and HBV. This gap highlights a need for comprehensive validation studies focusing on other STBBIs, such as HSV, HTLV, and syphilis. The latter is of particular concern due to the significant uptick in syphilis cases globally and in Canada [36–38].

DBS specimens offer a promising alternative to plasma and serum samples for STBBI testing, though assay performance can be dependent on several factors. We identified multiple potential factors which could influence test performance with DBS specimens. Adjustment of test cut-off values for DBS specimens improved overall performance [39], making it imperative for individual laboratories (or manufacturers) to validate specific cut-off values for use with DBS specimens. The presence of dual infections within a population also influenced assay performance [29], resulting in decreased sensitivity, especially for HBV and HCV testing in participants living with HIV. This finding is relevant for future test development and validation for future surveillance in key populations where dual infections may be more prevalent and in geographies which experience overlapping STBBI burdens [40]. However, we cannot draw larger conclusions regarding the impact of dual infections on test performance due to our inability to conduct a meta analysis. If assay performance is indeed impacted by dual infections, this could lead to underestimates of coinfections in key populations (for example: HCV seroprevalence in people who inject drugs and are living with HIV). Approaches to DBS preparation (venous versus capillary blood) also affected test performance [41]. Although DBS prepared from venous blood appears to offer better test performance (especially for HIV nucleic acid testing), DBS prepared from capillary blood still offer excellent test performance and reflects more closely “real-world” DBS collection, especially considering self-collection. In the context of serosurveillance, it is unlikely that DBS would be prepared with venous blood. Some studies may validate assays using DBS prepared from venous blood out of convenience. Though the observed difference between preparation methods is small, it highlights how sample preparation at the validation stage has the potential to influence assay performance and, by extension, prevalence estimates once an assay is implemented for surveillance activities. Enhanced reporting of DBS validation studies could lead to further improvements in the performance of DBS testing using capillary blood. Finally, test performance may fluctuate depending on the duration of ART among people living with HIV. Our review supports the validity of using DBS specimens for detecting certain STBBIs. DBS specimens perform well particularly well in HIV and HCV testing, though no conclusions can be made for chlamydia or gonorrhea surveillance as no studies investigated DBS testing with either pathogen. However, test performance relies on several experimental conditions, and standardized approaches to reporting DBS experiments should be adopted moving forward to ensure internal and external test validity.

Due to considerable heterogeneity observed across studies, we could not conduct a meta-analysis. General poor reporting of experimental conditions also created challenges in directly comparing studies–this was also reflected in our quality assessment using the QUADAS-2 tool. There is currently no consensus on how DBS studies should be reported. We propose that new standardized guidelines for reporting DBS experiments should be developed and implemented similarly to the MIQE guidelines for quantitative real-time PCR experiments [42] or the STARD checklist [43]. Reporting guidelines should include the minimum information required to evaluate DBS studies, including information on DBS preparation (venous or capillary blood), drying and storage conditions, type of filter paper used, and DBS elution protocols (for example volume, type of buffer, and agitation conditions). This would allow for a more direct comparison of studies and assist in conducting robust meta-analyses to further investigate the validity of DBS specimens for STBBI testing. Though we did not record storage temperature and humidity in this review, these factors have been examined by Amini et al. 2021 [44] in a systematic review on reliability of antibody measurement in DBS specimens. Though their objective differs from our review and includes fewer papers (*n* = 40), the similar observations of heterogeneity in experimental conditions and reporting further our observation that it is necessary to record a large number of influential variables to allow for standardized assessment of studies.

While treatment failure was not explicitly part of our objective, it is an important component of surveillance. More specifically, it can provide insights into the cascade of care and progress toward HIV and HCV elimination targets [45, 46]. Viral load testing alone is not sufficient for the purposes of surveillance as persons undergoing treatment may present undetectable viral loads, and therefore must be used in combination with serological assays to establish disease status. We decided to include studies investigating viral load with DBS specimens as viral load data still provide valuable insight into the cascade of care or other metrics towards elimination. It should be noted that the LOD for most HIV tests included in this review was determined to be approximately 800 copies/mL. The WHO defines virological suppression as <1,000 copies/mL, which should be reliably detected by most assays in this review [47, 48]. This finding is in agreement with a recent systematic review conducted by Vojnov et al. (2022) which concluded DBS specimens are suitable for HIV viral load testing at the treatment failure threshold of 1,000 copies/mL [14]. Although we consider DBS specimens suitable for certain STBBI surveillance, careful attention should be paid to the LOD of each assay under consideration and consider definitions of treatment failure for the STBBI of interest.

Limitations of this review include the unavailability of any studies on DBS testing for chlamydia and gonorrhea. We limited our biological specimen of interest to blood, and therefore did not include any papers on dried urine spots for chlamydia or gonorrhea detection. While the observed high sensitivity and specificity across studies provide evidence for the suitability of DBS for the surveillance of STBBIs, the majority of these findings (approximately 90%) pertain to HIV, HCV, and HBV. The limited data on syphilis, HTLV, HAV, HSV, and HPV introduces some uncertainty concerning the validity of DBS for the surveillance of these infections. Consequently, further validation studies are likely needed to ascertain if DBS can be recommended for these STBBIs with the same level of confidence as for HIV, HCV, and HBV. Additionally, we were unable to conduct a meta-analysis as a result of the large amount of heterogeneity between studies. We were unable to critically examine the influence of certain factors such as choice of elution protocol or reference test due to the high level of variation observed in these areas in the included studies. Finally, we did not examine acceptability of DBS testing among participants versus traditional methods, as this is beyond the scope of this review and warrants its own investigation.

## Conclusions

Over the course of the COVID-19 pandemic, the use of DBS specimens increased in popularity as many studies adopted them for serological studies [10, 49–51]. We anticipate that this increase in DBS usage will carry forward and result in a greater number of studies using DBS specimens for STBBI testing. Our review not only highlights the validity of DBS sampling for HIV, HCV, and HBV testing but also emphasises the current need for further validation studies for other STBBIs, including syphilis, HTLV, HAV, HSV, and HPV. More importantly, our review identifies a need for standardized reporting of experimental detail for studies involving the use of DBS specimens to ensure optimal and consistent performance across various settings. The use of DBS sampling is particularly promising for enhancing STBBI surveillance efforts in Canada’s remote and underserved communities, as well as in other resource-limited settings globally. By reducing barriers to biological specimen collection, DBS specimens stand as an important approach towards achieving and monitoring progress against global STBBI elimination targets, including the Joint United Nations Programme on HIV/AIDS (UNAIDS) 95-95-95 targets for HIV and the World Health Organization’s 2030 viral hepatitis elimination goals.

## Supporting information

S1 Text Search strategy MeSH terms and keywords. (DOCX)

S1 Data Characteristics of included studies. (XLSX)

S1 Table Studies reporting limit of detection and/or limit of quantification with DBS specimens. (DOCX)

S2 Table Studies reporting test performance according to test cut-off values. (DOCX)

S3 Table Studies comparing DBS prepared from venous and capillary blood. (DOCX)

S4 Table Studies assessing test performance on DBS specimens collected from participants with co-infections. (DOCX)

S5 Table Studies assessing test performance on DBS specimens collected from participants undergoing ART and ART-naïve participants. (DOCX)

S1 Checklist PRISMA 2020 checklist. (PDF)
