# Supplementary material for: Validity of dried blood spot testing for sexually transmitted and blood-borne infections: A narrative systematic review
Source: PLOS Glob Public Health. 2024 Jun 14;4(6):e0003320. doi: 10.1371/journal.pgph.0003320 (PMC11178196; doi:10.1371/journal.pgph.0003320)
Supplement: S1 Table — (DOCX) [file pgph.0003320.s003.docx]

**S1 Table**

*Studies reporting limit of detection and/or limit of quantification with DBS specimens*

| Study | Index Test | STBBI | LOD | LOQ | Notes |
| --- | --- | --- | --- | --- | --- |
| Aitken *et al*. 2013 | In-house RT-qPCR | HIV | 1,000 copies/mL | NR | DBS stored at ambient temperature (2 to 192 days) |
|  | In-house RT-qPCR | HIV | 1,000 copies/mL | NR | DBS stored at -20°C (45 to 112 days) |
|  | In-house RT-qPCR | HIV | 1,000 copies/mL | NR | DBS stored at -70°C (270 to 515 days) |
| Alvarez *et al.* 2015 | COBAS AmpliPrep/TaqMan HIV-1 Quantitative Test v2.0 (Roche) | HIV | 400 copies/mL | NR | Mothers and infants (ART naïve) |
|  | COBAS AmpliPrep/TaqMan HIV-1 Quantitative Test v2.0 (Roche) | HIV | 400 copies/mL | NR | Mothers and infants (on ART) |
|  | COBAS AmpliPrep/TaqMan HIV-1 Quantitative Test v2.0 (Roche) | HIV | 400 copies/mL | NR | Mothers and infants (all) |
|  | Versant HIV-1 RNA 1.0 (Siemens) | HIV | ~880 copies/mL | NR | Mothers and infants (ART naïve) |
|  | Versant HIV-1 RNA 1.0 (Siemens) | HIV | ~880 copies/mL | NR | Mothers and infants (on ART) |
|  | Versant HIV-1 RNA 1.0 (Siemens) | HIV | ~880 copies/mL | NR | Mothers and infants (all) |
| Andreotti *et al.* 2010 | COBAS TaqMan RT-qPCR (Roche) | HIV | 313 copies/mL | NR | Pregnant women (ART naïve) |
|  | COBAS TaqMan RT-qPCR (Roche) | HIV | 313 copies/mL | NR | Pregnant women (on ART) |
| Choudhary *et al.* 2013 | In-house PCR | HIV | 1,300 CEM cells | NR | HIV-1 proviral test |
| Erba *et al.* 2015 | m2000sp, m2000rt (Abbott) | HIV | 616 copies/mL | NR |  |
| Fiscus *et al.* 1998 | NASBA HIV-1 QT (Organon Teknika) | HIV | 1,000 copies/mL | NR |  |
| Huang *et al.* 2011 | RealTime HIV-1 Qualitative Assay (Abbott) | HIV | 2,469 copies/mL | NR | DBS prepared with material from the Virology Quality Assurance Laboratory (AIDS Clinical Trial Group) |
|  | RealTime HIV-1 Qualitative Assay (Abbott) | HIV | 3,085 copies/mL | NR | DBS prepared from the WHO 2^nd^ International Standard |
| Kane *et al.* 2008 | NucliSENS HIV-1 EasyQ (bioMérieux) | HIV | 870 IU/mL | NR |  |
| Marconi *et al.* 2009 | m2000sp, m2000rt (Abbott) | HIV | 200 – 1,000 copies/mL | NR |  |
| Nugent *et al.* 2009 | Aptima HIV-1 RNA Qualitative Assay (Gen-Probe) | HIV | 2,384 copies/mL | NR |  |
| Tang *et al.* 2017 | m2000 RealTime HIV-1 RNA (Abbott) | HIV | 839 copies/mL | 839 copies/mL |  |
| Templer *et al.* 2016 | TaqMan HIV-1 v2.0 (Roche) | HIV | 221.8 copies/mL | NR |  |
|  | TaqMan HIV-1 v1.0 (Roche) | HIV | 1,090 copies/mL | NR |  |
|  | RealTime HIV-1 Qualitative (Abbott) | HIV | 2,500 copies/mL | NR |  |
| Van Deursen *et al.* 2010 | NucliSENS EasyQ HIV-1 v2.0 (bioMérieux) | HIV | 800 copies/mL | NR |  |
| Viljoen *et al.* 2010 | Generic HIV Viral Load (Biocentric) | HIV | 3,100 copies/mL | NR | Durban (infants) |
|  | Generic HIV Viral Load (Biocentric) | HIV | 1,550 copies/mL | NR | Bobo-Dioulasso (mothers and infants) |
| Zeh *et al.* 2017 | m2000 RealTime HIV-1 RNA (Abbott) | HIV | 1,222 copies/mL | NR |  |
| Fajardo *et al.* 2014 | NucliSENS EasyQ HIV-1 v2.0 (bioMérieux) | HIV | NR | 100 copies/mL |  |
| Mavedzenge *et al.* 2015 | NucliSENS EasyQ HIV-1 v2.0 (bioMérieux) | HIV | NR | 100 copies/mL |  |
| Bennett *et al.* 2012 | In-house RT-qPCR | HCV | 250 IU/mL | NR |  |
| Catlett *et al.* 2019 | Aptima HCV Quant DX (Hologic) | HCV | 13 IU/mL | 525 IU/mL |  |
| De Crignis *et al.* 2010 | In-house RT-qPCR | HCV | 2,500 copies/mL | NR |  |
| Mahajan *et al.* 2018 | m2000rt HCV (Abbott) | HCV | 1,000 IU/mL | NR |  |
| Marques *et al.* 2016 | In-house RT-qPCR | HCV | 58.5 copies/mL | NR |  |
| Nguyen  *et al.* 2018 | Generic HCV Assay (Biocentric) | HCV | 3,000 IU/mL | NR |  |
| Vazquez-Moron *et al.* 2018 | In-house RT-qPCR | HCV | 5,000 copies/mL | NR |  |
| Weber *et al.* 2019 | Aptima HCV Quant DX (Hologic) | HCV | 267 IU/mL | NR |  |
| Parr *et al.* 2018 | m2000 RealTime HCV Viral Load (Abbott) | HCV | 1,196 IU/mL | NR | 6 mm DBS punches |
|  | m2000 RealTime HCV Viral Load (Abbott) | HCV | 494 IU/mL | NR | 12 mm DBS punches |
| Santos *et al.* 2012 | In-house RT-qPCR | HCV | 50 IU/mL | NR |  |
| Shepherd *et al.* 2019 | m2000 RealTime HCV Viral Load (Abbott) | HCV | 178 – 1,779 IU/mL | NR |  |
| Solmone *et al.* 2002 | In-house RT-qPCR | HCV | 960 IU/mL | NR |  |
| Saludes *et al.* 2018 | In-house RT-qPCR | HCV | 541 UI/mL | NR |  |
| Stene-Johansen *et al.* 2016 | In-house qPCR | HBV | 501.19 IU/mL | NR |  |
| Jardi *et al.* 2004 | In-house qPCR | HBV | 2,000 copies/mL | NR |  |
| Mohamed *et al.* 2013 | Chemiluminescent microparticle immunoassay (Abbott) | HBV | 0.30 ± 0.08 IU/mL | NR | HBAg |
|  | Chemiluminescent microparticle immunoassay (Abbott) | HBV | 18.11 ± 6.05 IU/mL | NR | Anti-HBs |
|  | AmpliPrep/COBAS TaqMan HBV Test v2.0 (Roche) | HBV | 914.1 ± 157.8 IU/mL | NR | HBV DNA |
| Vinikoor *et al.* 2015 | AmpliPrep/COBAS TaqMan HBV v2.0 (Roche) | HBV | 1,000 IU/mL | NR |  |
| Wilson *et al.* 2018 | ARCHITECT Qualitative HBsAg Assay (Abbott) | HBV | 0.75 IU/mL | NR |  |
| Lira *et al.* 2009 | HBV Monitor COBAS Amplicor v1.5 (Roche) | HBV | 2,000 copies/mL | NR |  |
| Zhang *et al.* 2010 | In-house PCR | HBV | 5 copies/mL | NR |  |
| Jackson *et al.* 2022 | RealTime HBV Viral Load (Abbott) | HBV | 389 IU/mL | NR |  |
| Roger *et al.* 2020 | Aptima HBV Quant DX (Hologic) | HBV | 445 IU/mL | NR |  |
| Shimakawa *et al.* 2021 | LUMIPULSE CLEIA (Fujirebio) | HBV | 19,115 IU/mL | NR | HBV core-related antigen |
| Bezerra *et al.* 2022 | In-house qPCR | HBV | 852.5 copies/mL | NR |  |
| Bezerra *et al.* 2021 | In-house qPCR | HBV | 20 copies/mL | NR | High Pure Viral Nuleic Acid Kit (Roche) |
| Bargain *et al.* 2020 | GeneXpert HBV (Cepheid) | HBV | NR | 400 IU/mL |  |
| Noda *et al.* 1993 | In-house PCR | HTLV | Single HUT102 cell | NR |  |

ART = antiretroviral therapy; DBS = dried blood spot; IU/mL = international units/mL; LOD = limit of detection; LOQ = limit of quantification; NR = not reported; STBBI = sexually transmitted and blood-borne infection
