# Supplementary material for: Validity of dried blood spot testing for sexually transmitted and blood-borne infections: A narrative systematic review
Source: PLOS Glob Public Health. 2024 Jun 14;4(6):e0003320. doi: 10.1371/journal.pgph.0003320 (PMC11178196; doi:10.1371/journal.pgph.0003320)
Supplement: S2 Table — (DOCX) [file pgph.0003320.s004.docx]

**S2 Table**

*Studies reporting test performance according to test cut-off values*

| Study | STBBI | Index Test | Cut-Off | Sensitivity  (95% CI) | Specificity  (95% CI) | PPV  (95% CI) | NPV  (95% CI) | Notes |
| --- | --- | --- | --- | --- | --- | --- | --- | --- |
| García-Cisneros *et al.* 2019 | HSV-2 | IgG-G2 Human (Human Diagnostics) | ≥1.15^a^ | 99.7  (98.3-100.0) | 4.5  (3.0-6.5) | 37.3  (34.1-40.6) | 96.3  (81.0-99.9) |  |
|  |  |  | ≥4.61^b^ | 90.4  (84.4-94.4) | 87.1  (81.2-91.4) | 84.7  (77.9-89.8) | 92.0  (86.7-95.4) |  |
| Villar *et al.* 2011 | HBV | ETI-AB-COREK Plus  (DiaSorin) | Mean of 3 calibrators x 0.3^a^ | 89.2  (79.8-95.2) | 97.5  (91.4-99.7) | 97.1  (89.8-99.6) | 90.8  (82.7-96.0) |  |
|  |  |  | ≤0.951^b^ | 100.0  (95.1-100.0) | 2.5  (0.3-8.6) | 48.4  (40.2-56.6) | 100.0  (15.8-100.0) |  |
|  |  |  | ≤0.261^b^ | 90.5  (81.5-96.1) | 92.6  (84.6-97.2) | 91.8  (83.0-96.9) | 91.5  (83.2-96.5) |  |
|  |  | ETI-AB-AUK-3 (DiaSorin) | Mean of kit calibrator 1^a^ | 83.1  (71.0-91.6) | 81.3  (70.7-89.4) | 77.8  (65.5-87.3) | 85.9  (75.6-93.0) |  |
|  |  |  | >0.119^b^ | 66.1  (52.6-77.6) | 98.7  (92.8-100.0) | 97.5  (86.8-99.9) | 78.7  (69.1-86.5) |  |
|  |  |  | >0.095^b^ | 78.0  (65.3-87.7) | 97.3  (90.7-99.7) | 95.8  (85.8-99.5) | 84.9  (75.5-91.7) |  |
|  |  | ETI-MAK-4 (DiaSorin) | Mean of negative control + 0.03^a^ | 95.5  (84.5-99.4) | 70.8  (60.2-80.0) | 61.8  (49.2-73.3) | 96.9  (89.3-99.6) |  |
|  |  |  | >0.100^b^ | 93.2  (81.3-98.6) | 98.9  (93.9-100.0) | 97.6  (87.4-99.9) | 96.7  (90.7-99.3) |  |
|  |  |  | >0.115^b^ | 97.6  (87.4-99.9) | 96.7  (90.7-99.3) | 93.2  (81.3-98.6) | 98.9  (93.9-100.0) |  |
| Catlett *et al.* 2019 |  | Aptima HCV Quant Dx (Hologic) | Detected | 96.4  (89.8-99.3) | 95.8  (78.9-99.9) | NR | NR |  |
|  |  |  | ≥15 IU/mL | 95.1  (88.0-98.7) | 96.0  (79.7-99.9) | NR | NR |  |
|  |  |  | ≥1,000 IU/mL | 100 .0  (95.3-100.0) | 100.0  (88.4-100.0) | NR | NR |  |
| Brandão *et al.* 2013 | HCV | Monolisa HCV AgAb ULTRA EIA (Bio-Rad) | Not specified^a^ | 95.0  (83.1-99.4) | 100.0  (98.9-100.0) | 100.0  (90.8-100.0) | 99.4  (97.9-99.9) |  |
|  |  |  | 0.108^b^ | 97.5  (86.8-99.9) | 97.2  (94.9-98.6) | 79.6  (65.7-89.8) | 99.7  (98.4-99.0) |  |
|  |  |  | 0.287^b^ | 97.5  (86.8-99.9) | 99.7  (98.4-100.0) | 100.0  (91.0-100.0) | 100.0  (99.0-100.0) |  |
|  |  | Murex HCV AgAb Combination EIA (DiaSorin) | Not specified^a^ | 82.5  (67.2-92.7) | 98.0  (95.9-99.2) | 82.5  (67.2-92.7) | 98.0  (95.9-99.2) |  |
|  |  |  | 0.514^b^ | 80.0  (64.3-90.9) | 99.4  (97.9-99.9) | 94.1  (80.3-99.3) | 97.7  (95.6-99.0) |  |
|  |  |  | 0.239^b^ | 97.5  (86.8-99.9) | 96.0  (93.3-97.8) | 73.6  (93.3-97.8) | 100  (98.3-100.0) |  |
| Erba *et al.* 2015 | HIV | m2000SP, m2000RT (Abbott) | ≥40 copies/mL | 74.0  (66.9-80.3) | 99.0  (94.5-99.8) | 99.2  (95.8-99.9) | 68.3  (60.0-75.8) |  |
|  |  |  | ≥1,000 copies/mL | 94.2  (88.9-97.5) | 98.6  (94.9-99.8) | 98.5  (94.6-99.8) | 94.5  (89.4-97.6) |  |
| Fajardo *et al.* 2014 | HIV | NucliSENS EasyQ HIV-1 v2.0 (bioMérieux) | ≥1,000 copies/mL | 88.7  (81.1-94.0) | 97.8  (96.1-98.9) | NR | NR | DBS prepared from capillary blood (finger pokes) |
|  |  |  | ≥3,000 copies/mL | 84.9  (76.0-91.5) | 99.8  (98.9-100.0) | NR | NR | DBS prepared from capillary blood (finger pokes) |
|  |  |  | ≥5,000 copies/mL | 83.0  (73.4-90.1 | 100.0  (99.3-100.0) | NR | NR | DBS prepared from capillary blood (finger pokes) |
|  |  |  | ≥1,000 copies/mL | 91.4  (84.4-96.0) | 97.2  (95.4-98.5) | NR | NR | DBS prepared from venous blood |
|  |  |  | ≥3,000 copies/mL | 90.0  (82.2-95.4) | 99.4  (98.3-99.9) | NR | NR | DBS prepared from venous blood |
|  |  |  | ≥5,000 copies/mL | 88.5  (79.9-94.3) | 99.8  (98.9-100.0) | NR | NR | DBS prepared from venous blood |
| Guichet *et al.* 2018 | HIV | In-house RT-qPCR | ≥1,000 copies/mL | 90.0 (83.0-96.0) | 49.0 (37.0-61.0) | NR | NR | NucliSENS (bioMérieux) |
|  |  |  | ≥5,000 copies/mL | 74.0  (62-86.0) | 77.0  (69.0-85.0) | NR | NR | NucliSENS (bioMérieux) |
|  |  |  | ≥1,000 copies/mL | 80.0  (71.0-89.0) | 59.0  (47.0-71.0) | NR | NR | m2000SP (Abbott) |
|  |  |  | ≥5,000 copies/mL | 79.0  (68.0-90.0) | 68.0  (59.0-72.0) | NR | NR | m2000SP (Abbott) |
|  |  |  | ≥1,000 copies/mL | 80.0  (71.0-89.0) | 100.0  (100.0-100.0) | NR | NR | NucliSENS (bioMérieux), Turbo DNase-Free (Ambion) |
|  |  |  | ≥5,000 copies/mL | 23.0  (12.0-34.0) | 100.0  (100.0-100.0) | NR | NR | NucliSENS (bioMérieux), Turbo DNase-Free (Ambion) |
|  |  |  | ≥1,000 copies/mL | 27.0  (17.0-37.0) | 81.0  (70.0-90.0) | NR | NR | NucliSENS (bioMérieux), HL-dsDNase (TATAA Biocenter AB) |
|  |  |  | ≥5,000 copies/mL | 51.0  (37.0-65.0) | 92.0  (86.0-97.0) | NR | NR | NucliSENS (bioMérieux), HL-dsDNase (TATAA Biocenter AB) |
|  |  |  | ≥1,000 copies/mL | 60.0  (49.0-71.0) | 82.0  (73.0-91.0) | NR | NR | NucliSENS (bioMérieux), FVE protocol |
|  |  |  | ≥5,000 copies/mL | 49.0  (35.0-62.0) | 99.0  (97.0-100.0) | NR | NR | NucliSENS (bioMérieux), FVE protocol |
|  |  |  | ≥1,000 copies/mL | 83.0  (75.0-91.0) | 58.0  (46.0-70.0) | NR | NR | m2000SP, m2000RT (Abbott) |
|  |  |  | ≥5,000 copies/mL | 75.0  (63.0-87.0) | 89.0  (83.0-95.0) | NR | NR | m2000SP, m2000RT (Abbott) |
| Halfon *et al.* 2012 | HBV | HBV COBAS TaqMan (Roche) | ≥1,400 IU/mL | 98.0  (95.0-100.0) | 100.0  (100.0-100.0) | NR | NR |  |
|  |  |  | ≥2,000 IU/mL | 91.0  (85.0-97.0) | 100.0  (100.0-100.0) | NR | NR |  |
| Hobbs *et al.* 2017 | HSV-2 | Kalon HSV-2 ELISA (Kalon Biological Ltd) | >1.1^a^ | 98.8  (92.7-99.9) | 98.9  (93.4-99.9) | NR | NR |  |
|  |  |  | >1.5^b^ | 98.8  (93.6-99.8) | 98.9  (94.2-99.8) | NR | NR |  |
| Judd *et al.* 2003 | HCV | Ortho HCV 3.0 (Bio-Rad) | 0.100^a^ | 99.2 | 100.0 |  |  |  |
|  |  |  | Negative mean + 1 SD^b^ | 100.0 | 92.4 | NR | NR |  |
|  |  |  | Negative mean + 2 SD^b^ | 100.0 | 96.9 | NR | NR |  |
|  |  |  | Negative mean + 3 SD^b^ | 99.6 | 99.7 | NR | NR |  |
|  |  |  | Negative mean + 4 SD^b^ | 99.6 | 99.7 | NR | NR |  |
|  |  |  | Negative mean + 5 SD^b^ | 99.6 | 100.0 | NR | NR |  |
|  |  |  | Negative mean + 6 SD^b^ | 99.2 | 100.0 | NR | NR |  |
| Makadzange *et al.* 2017 | HIV | COBAS AmpliPrep/COBAS TaqMan (Roche) | Detected | 77.0  (69.4-85.5) | 100.0  (97.1-100.0) | 100.0  (96.8-100.0) | 78.5  (71.2-84.6) |  |
|  |  |  | ≥1,000 copies/mL | 92.7  (88.6-96.6) | 100.0  (97.6-100.0) | 100.0  (96.8-100.0) | 94.3  (89.5-97.4) |  |
|  |  |  | ≥5,000 copies/mL | 70.9  (61.8-79.0) | 100.0  (97.6-100.0) | 100.0  (95.7-100.0) | 82.0  (75.8-87.2) |  |
| Marques *et al.* 2012 | HCV | ETI-AB-HCVK-4 (DiaSorin) | 0.592^a^ | 88.9  (76.0-96.3) | 96.1  (93.6-97.9) | 74.1  (60.4-85.0) | 98.6  (96.7-99.5) |  |
|  |  |  | 0.648^b^ | 88.9  (76.0-96.3) | 96.4  (93.9-98.1) | 75.5  (61.7-86.2) | 98.6  (96.7-99.5) |  |
|  |  |  | 1.345^b^ | 88.9  (76.0-96.3) | 98.6  (96.8-99.6) | 88.9  (76.0-96.3) | 98.6  (96.8-99.6) |  |
|  |  | HCV Ab (Radim) | 0.347^a^ | 97.5  (86.8-99.9) | 99.5  (98.1-99.9) | 95.1  (83.5-99.4) | 99.7  (98.5-100.0) |  |
|  |  |  | 0.351^b^ | 97.5  (86.8-99.9) | 99.5  (98.1-99.9) | 95.1  (83.5-99.4) | 99.7  (98.5-100.0) |  |
|  |  |  | 0.284^b^ | 97.5  (86.8-99.9) | 99.2  (97.7-99.8) | 92.9  (80.5-98.5) | 99.7  (98.5-100.0) |  |
| McCarron *et al.* 1999 | HCV | Monolisa anti-HCV (Sanofi Pasteur) | >0.99^b^ | 100.0 | 87.5 | NR | NR |  |
|  |  |  | >1.99^b^ | 97.2 | 100.0 | NR | NR |  |
| Neogi *et al.* 2012 | HIV | m2000RT, m2000SP (Abbott) | 2.17 to 3 log^10^ copies/mL | 50.0 | 100.0 | NR | NR |  |
|  |  |  | >3 to 3.7 log^10^ copies/mL | 90.0 | 100.0 | NR | NR |  |
|  |  |  | >3.7 log^10^ copies/mL | 100.0 | 100.0 | NR | NR |  |
| Nugent *et al.* 2009 | HIV | Aptima HIV-1 RNA Qualitative Assay (Gen-Probe) | ≥500 copies/mL | 65.0 | NR | NR | NR |  |
|  |  |  | ≥2,500 copies/mL | 92.0 | NR | NR | NR |  |
|  |  |  | ≥5,000 copies/mL | 100 | NR | NR | NR |  |
|  |  |  | ≥10,000 copies/mL | 100 | NR | NR | NR |  |
| Pannus *et al.* 2013 | HIV | NucliSENS EasyQ v2.0 (bioMérieux) | ≥1,000 copies/mL | 78.6  (59.0-91.7) | 100.0  (98.9-100.0) | 100.0  (84.6-100.0) | 98.2  (96.1-99.3) | DBS prepared from capillary blood (finger pokes) |
|  |  |  | ≥1,000 copies/mL | 89.3  (71.8-97.7) | 99.7  (98.3-100.0) | 96.2  (80.4-99.9) | 99.1  (97.3-99.8) | DBS prepared from venous blood |
|  |  |  | ≥5,000 copies/mL | 69.6  (47.1-86.8) | 100.0  (89.9-100.0) | 100.0  (79.4-100.0) | 97.9  (95.7-99.2) | DBS prepared from capillary blood (finger pokes) |
|  |  |  | ≥5,000 copies/mL | 60.9  (38.5-80.3) | 100.0  (98.9-100.0) | 100.0  (76.8-100.0) | 97.3  (95.0-98.8) | DBS prepared from venous blood) |
| Pirillo *et al.* 2011 | HIV | VERSANT HIV-1 RNA 1.0 (Siemens) | ≥37 copies/mL | 88.2  (79.4-93.6) | 69.2  (42.0-87.4) | 94.9  (90.1-99.8) | 47.4  (24.9-69.8) |  |
|  |  |  | ≥5,000 copies/mL | 85.1  (76.5-88.6) | 96.1  (88.2-99.3) | 95.2  (85.6-99.1) | 87.5  (80.3-90.4) |  |
| Pollack *et al.* 2018 | HIV | COBAS AmpliPrep/COBAS TaqMan (Roche) | ≥1,000 copies/mL | 98.8  (93.3-100.0) | 74.3  (70.8-77.5) | 31.5  (25.8-37.6) | 99.8  (98.9-100.0) | Guanidinium pre-extraction |
|  |  |  | ≥5,000 copies/mL | 92.4  (84.2-97.2) | 97.9  (96.6-98.9) | 83.9  (74.5-90.9) | 99.1  (98.1-99.7) | Guanidinium pre-extraction |
|  |  |  | ≥1,000 copies/mL | 95.1  (87.8-98.6) | 98.8  (97.7-99.5) | 90.6  (82.3-95.8) | 99.4  (98.5-99.8) | Correction factor applied (0.3 log^10^ copies/mL) |
|  |  |  | ≥5,000 copies/mL | 96.3  (89.6-99.2) | 98.2  (96.9-99.1) | 86.7  (77.9-92.9) | 99.6  (98.7-99.9) | Correction factor applied (0.3 log^10^ copies/mL) |
|  |  |  | ≥1,000 copies/mL | 65.8  (54.3-76.1) | 100.0  (99.5-100.0) | 100.0  (93.2-100.0) | 96.2  (94.5-97.5) | Correction factor applied (0.7 log^10^ copies/mL) |
|  |  |  | ≥5,000 copies/mL | 84.8  (75.0-91.9) | 99.7  (98.9-100.0) | 97.1  (89.9-99.6) | 98.3  (97.0-99.1) | Correction factor applied (0.7 log^10^ copies/mL) |
| Rutstein *et al.* 2014 | HIV | m2000RT, m2000SP (Abbott) | ≥1,000 copies/mL | 100.0 | 97.1 | 75.0 | 100.0 | DBS prepared from venous blood |
|  |  |  | ≥1,000 copies/mL | 100.0 | 94.9 | 63.2 | 100.0 | DBS prepared from capillary blood (finger pokes) |
|  |  |  | ≥5,000 copies/mL | 100.0 | 98.6 | 83.3 | 100.0 | DBS prepared from venous blood |
|  |  |  | ≥5,000 copies/mL | 100.0 | 97.8 | 76.9 | 100.0 | DBS prepared from capillary blood (finger pokes) |
| Sawadogo *et al.* 2014 | HIV | COBAS AmpliPrep/COBAS TaqMan (Roche) | ≥1,000 copies/mL | 99.0  (97.0-100.0) | 26.0  (22.0-29.0) | 29.0  (26.0-33.0) | 99.0  (96.0-100.0) |  |
|  |  |  | ≥5,000 copies/mL | 99.0  (96.0-100.0) | 55.0  (51.0-59.0) | 33.0  (29.0-37.0) | 100.0  (98.0-100.0) |  |
| Schmitz *et al.* 2017 | HIV | COBAS AmpliPrep/COBAS TaqMan (Roche) | ≥1,000 copies/mL | 90.1  (85.7-936) | 93.1  (90.6-95.2) | NR | NR | DBS prepared from venous blood |
|  |  | COBAS AmpliPrep/COBAS TaqMan (Roche) | ≥1,000 copies/mL | 90.3  (85.7-93.7) | 94.9  (92.6-96.6) | NR | NR | DBS prepared from capillary blood (microtainer) |
|  |  | COBAS AmpliPrep/COBAS TaqMan (Roche) | ≥1,000 copies/mL | 88.1  (83.3-92.0) | 94.5  (92.1-96.3) | NR | NR | DBS prepared from capillary blood (finger pokes) |
|  |  | RealTime HIV-1 (Abbott) | ≥1,000 copies/mL | 94.4  (90.6-97.0) | 33.0  (28.9-37.3) | NR | NR | DBS prepared from venous blood |
|  |  | COBAS AmpliPrep/COBAS TaqMan (Roche) | ≥3,000 copies/mL | 85.2  (80.1-89.4) | 98.0  (96.4-99.1) | NR | NR | DBS prepared from venous blood |
|  |  | COBAS AmpliPrep/COBAS TaqMan (Roche) | ≥3,000 copies/mL | 88.1  (83.3-92.0) | 97.6  (95.9-98.8) | NR | NR | DBS prepared from capillary blood (microtainer) |
|  |  | COBAS AmpliPrep/COBAS TaqMan (Roche) | ≥3,000 copies/mL | 85.2  (80.0-89.4) | 97.8  (96.2-98.9) | NR | NR | DBS prepared from capillary blood (finger pokes) |
|  |  | RealTime HIV-1 (Abbott) | ≥3,000 copies/mL | 88.4  (83.5-92.2) | 60.9  (56.6-65.2) | NR | NR | DBS prepared from venous blood |
|  |  | COBAS AmpliPrep/COBAS TaqMan (Roche) | ≥5,000 copies/mL | 83.1  (77.8-87.6) | 98.4  (96.9-99.3) | NR | NR | DBS prepared from venous blood |
|  |  | COBAS AmpliPrep/COBAS TaqMan (Roche) | ≥5,000 copies/mL | 83.9  (78.6-88.3) | 98.6  (97.2-99.4) | NR | NR | DBS prepared from capillary blood (microtainer) |
|  |  | COBAS AmpliPrep/COBAS TaqMan (Roche) | ≥5,000 copies/mL | 82.2  (76.7-86.9) | 98.8  (97.4-99.6) | NR | NR | DBS prepared from capillary blood (finger pokes) |
|  |  | RealTime HIV-1 (Abbott) | ≥5,000 copies/mL | 79.7  (74.0-84.7) | 77.0  (73.1-80.5) | NR | NR | DBS prepared from venous blood |
| Taieb *et al.* 2018 | HIV | m2000SP, m2000RT (Abbott) | Detected | 95.8  (88.1-99.1) | 63.6  (54.8-71.8) | NR | NR |  |
|  |  |  | ≥839 copies/mL | 91.5  (82.5-96.8) | 95.5  (90.3-98.3) | NR | NR |  |
|  |  |  | ≥1,000 copies/mL | 90.1  (80.7-95.9) | 96.2  (91.4-98.8) | NR | NR |  |
|  |  |  | ≥3,000 copies/mL | 84.5  (74.0-92.0) | 99.2  (95.9-100.0) | NR | NR |  |
|  |  |  | ≥5,000 copies/mL | 80.3  (69.1-88.8) | 100.0  (97.2-100.0) | NR | NR |  |
| Taieb *et al.* 2016 | HIV | COBAS AmpliPrep/COBAS TaqMan HIV-1 v2.0 (Roche) | ≥1,000 copies/mL | 54.9  (40.3-68.9) | 100.0  (97.5-100.0) | NR | NR |  |
|  |  |  | ≥3,000 copies/mL | 45.2  (29.8-61.3) | 100.0  (97.7-100.0) | NR | NR |  |
|  |  |  | ≥5,000 copies/mL | 47.5  (31.5-63.8) | 100.0  (97.7-100.0) | NR | NR |  |
|  |  | Real-Time HIV-1 (Abbott) | ≥1,000 copies/mL | 93.3  (81.7-98.6) | 94.8  (90.0-97.7) | NR | NR |  |
|  |  |  | ≥3,000 copies/mL | 97.4  (86.2-99.9) | 96.3  (92.0-98.6) | NR | NR |  |
|  |  |  | ≥5,000 copies/mL | 100  (90.5-100.0) | 98.8  (95.6-99.8) | NR | NR |  |
| Vidya *et al.* 2012 |  | m2000RT (Abbott) | ≤1,000 copies/mL | 62.0 | NR | NR | NR |  |
|  |  |  | 1,000-3,000 copies/mL | 88.0 | NR | NR | NR |  |
|  |  |  | >3,000 copies/mL | 100.0 | NR | NR | NR |  |
| Zeh *et al.* 2017 | HIV | COBAS AmpliPrep/COBAS TaqMan (Roche) | ≥400 copies/mL | 100.0  (97.6-100.0) | 4.0  (0.5-13.7) | 75.8  (69.2-81.6) | 100.0  (15.8-100.0) | Principal outcome was viral suppression (undetectable). HIV cell-associated DNA and RNA may have contributed to false detectable viral load readings in DBS samples. |
|  |  |  | ≥1,000 copies/mL | 100.0 | 17.3 | NR | NR |  |
|  |  |  | ≥2,000 copies/mL | 98.0 | 36.5 | NR | NR |  |
|  |  |  | ≥3,000 copies/mL | 97.3 | 54.0 | NR | NR |  |
|  |  |  | ≥4,000 copies/mL | 96.0 | 82.7 | NR | NR |  |
|  |  |  | ≥5,000 copies/mL | 95.3 | 84.6 | NR | NR |  |
|  |  | m2000RT (Abbott) | ≥550 copies/mL | 93.9  (88.8-97.2) | 88.0  (82.2-92.4) | 100.0  (97.4-100.0) | 85.3  (73.8-93.0) |  |
|  |  |  | ≥1,000 copies/mL | 96.6 | 90.4 | NR | NR |  |
|  |  |  | ≥2,000 copies/mL | 95.3 | 94.2 | NR | NR |  |
|  |  |  | ≥3,000 copies/mL | 94.6 | 98.1 | NR | NR |  |
|  |  |  | ≥4,000 copies/mL | 93.2 | 98.1 | NR | NR |  |
|  |  |  | ≥5,000 copies/mL | 93.0 | 98.1 | NR | NR |  |
| Biondi *et al.* 2019 | HCV | ARCHITECT Core Antigen (Abbott) | >3 fmol/L | 94.1  (88.5-99.7) | NR | NR | NR | DBS stored at -80°C |
|  |  |  | >10 fmol/L | 85.3  (76.4-94.2) | NR | NR | NR | DBS stored at -80°C |
|  |  |  | >3 fmol/L | 94.1  (88.5-99.7) | NR | NR | NR | DBS stored at 4°C |
|  |  |  | >10 fmol/L | 85.3  (76.4-94.2) | NR | NR | NR | DBS stored at 4°C |
|  |  |  | >3 fmol/L | 91.2  (84.3-98.1) | NR | NR | NR | DBS stored at ambient °C |
|  |  |  | >10 fmol/L | 80.9  (70.8-91.0) | NR | NR | NR | DBS stored at ambient °C |
|  |  |  | >3 fmol/L | 92.7  (86.4-98.9) | NR | NR | NR | DBS stored at 37°C |
|  |  |  | >10 fmol/L | 80.9  (70.8-91.0) | NR | NR | NR | DBS stored at 37°C |
|  |  |  | >3 fmol/L | 92.7  (86.4-98.9) | NR | NR | NR | DBS stored at 37°C followed by 4°C |
|  |  |  | >10 fmol/L | 85.3  (76.4-94.2) | NR | NR | NR | DBS stored at 37°C followed by 4°C |
| Catlett *et al.* 2019 | HCV | Aptima HCV Quant Dx (Hologic) | ≥12 IU/mL | 90.7  (80.0-97.0) | 100.0  (97.0-100.0) | NR | NR |  |
|  |  |  | ≥1,000 IU/mL | 92.5  (82.0-98.0) | 100.0  (97.0-100.0) | NR | NR |  |
|  |  |  | ≥3,000 IU/mL | 92.5  (92.0-98.0) | 100.0  (97.0-100.0) | NR | NR |  |
| Saludes *et al.* 2019 | HCV | In-house RT-qPCR | ~12 IU/mL | 88.3  (83.0-92.0) | NR | NR | NR |  |
|  |  |  | ≥12 IU/mL | 90.1  (85.0-93.5) | NR | NR | NR |  |
|  |  |  | ≥1,000 copies/mL | 96.1  (92.1-98.1) | NR | NR | NR |  |
|  |  |  | ≥3,000 copies/mL | 97.2  (93.5-98.8) | NR | NR | NR |  |
|  |  |  | ≥50,000 copies/mL | 100.0  (97.6-100.0) | NR | NR | NR |  |
| Saludes *et al.* 2020 | HCV | Xpert HCV VL Fingerstick (Cepheid) | ~10 IU/mL | 93.7  (84.8-97.5) | 100.0  (90.1-100.0) | NR | NR |  |
|  |  |  | ≥10 IU/mL | 96.7  (88.8-99.1) | 100.0  (90.1-100.0) | NR | NR |  |
|  |  |  | ≥1,000 IU/mL | 98.3  (91.1-99.7) | 100.0  (90.1-100.0) | NR | NR |  |
|  |  |  | ≥3,000 IU/mL | 98.3  (91.0-99.7) | 100.0  (90.1-100.0) | NR | NR |  |
| Tola *et al.* 2021 | HIV | m2000 RealTime HIV-1 (Abbott) | ≥1,000 copies/mL | 85.2  (81.1-89.3) | 91.5  (88.7-94.3) | NR | NR | DBS prepared from capillary blood (microtainer) |
|  |  |  | ≥3,000 copies/mL | 72.5  (67.3–77.7) | 96.9  (95.2–98.6) | NR | NR | DBS prepared from capillary blood (microtainer) |
|  |  |  | ≥5,000 copies/mL | 65.8  (60.3–71.4) | 97.2  (95.5–98.8) | NR | NR | DBS prepared from capillary blood (microtainer) |
|  |  |  | ≥1,000 copies/mL | 85.6  (81.5–89.6) | 90.9  (88.1–93.8) | NR | NR | DBS prepared from capillary blood (finger pokes) |
|  |  |  | ≥3,000 copies/mL | 71.5  (66.2–76.7) | 96.6  (94.9–98.4) | NR | NR | DBS prepared from capillary blood (finger pokes) |
|  |  |  | ≥5,000 copies/mL | 62.7  (57.1–68.3) | 97.4  (95.9–99.0) | NR | NR | DBS prepared from capillary blood (finger pokes) |
|  |  |  | ≥1,000 copies/mL | 89.1  (85.5–92.7) | 86.6  (83.2–90.0) | NR | NR | DBS prepared from venous blood |
|  |  |  | ≥3,000 copies/mL | 76.4  (71.5–81.3) | 96.1  (94.2–98.1) | NR | NR | DBS prepared from venous blood |
|  |  |  | ≥5,000 copies/mL | 67.2  (61.8–72.7) | 96.6  (95.2–98.6) | NR | NR | DBS prepared from venous blood |
| Kouamé *et al*. 2023 | HBV | HBsAg (version ULTRA) ELISA, (Dia.Pro Diagnostic Bioprobes S.R.L) | >Ratio of 1 (Ratio = OD/Threshold value) | 100 | 6.1 | NR | NR |  |
|  |  |  | >Ratio of 3 (Ratio = OD/Threshold value) | 100 | 47.0 | NR | NR |  |
|  |  |  | >Ratio of 5 (Ratio = OD/Threshold value) | 100 | 83.0 | NR | NR |  |
|  |  |  | >Ratio of 10 (Ratio = OD/Threshold value) | 100 | 100 | NR | NR |  |

^a^Manufacturer recommended

^b^Established in-house

NPV=negative predictive value; NR=not reported; PPV=positive predictive value; STBBI=sexually transmitted and blood-borne infection;
