# Supplementary material for: Validity of dried blood spot testing for sexually transmitted and blood-borne infections: A narrative systematic review
Source: PLOS Glob Public Health. 2024 Jun 14;4(6):e0003320. doi: 10.1371/journal.pgph.0003320 (PMC11178196; doi:10.1371/journal.pgph.0003320)
Supplement: S3 Table — (DOCX) [file pgph.0003320.s005.docx]

**S3 Table**

*Studies comparing DBS prepared from venous and capillary blood*

| Study | STBBI | Index Test | Preparation | Sensitivity  (95% CI) | Specificity  (95% CI) | PPV  (95% CI) | NPV  (95% CI) | Notes |
| --- | --- | --- | --- | --- | --- | --- | --- | --- |
| Prinsenberg *et al.* 2020 | HCV | COBAS AmpliPrep/COBAS TaqMan (Roche) | Capillary (finger poke) | 95.7  (78.1-99.9) | NR | NR | NR |  |
|  |  |  | Venous | 96.4  (81.7-99.9) | NR | NR | NR |  |
|  | HIV |  | Capillary (finger poke) | 100.0  (69.2-100.0) | NR | NR | NR |  |
|  |  |  | Venous | 100.0  (73.5-100.0) | NR | NR | NR |  |
| Tran *et al.* 2020 | HCV | m2000SP, m2000RT (Abbott) | Capillary (finger poke) | 98.6  (92.2-100.0) | NR | NR | NR | DBS stored at ambient °C (6-14 days) |
|  |  |  | Venous | 97.9  (92.7-99.7) | NR | NR | NR |  |
|  |  |  | Capillary (finger poke) | 97.8  (94.4-99.4) | NR | NR | NR | DBS stored at ambient °C (≥15 days) |
|  |  |  | Venous | 98.1  (94.4-99.6) | NR | NR | NR |  |
| Vetter *et al.* 2021 | HIV | INNOTEST HCV Ab IV (Fujirebio) | Capillary (finger poke) | 100.0  (97.5-100.0) | 100.0  (95.7-100.0) | NR | NR |  |
|  |  |  | Venous | 100.0  (97.5-100.0) | 100.0  (95.7-100.0) | NR | NR |  |
| Fajardo *et al.* 2014 | HIV | NucliSENS EasyQ HIV-1 v2.0 (bioMérieux) | Capillary (finger poke) | 88.7  (81.1-94.0) | 97.8  (96.1-98.9) | NR | NR | ≥1,000 copies/mL |
|  |  |  |  | 84.9  (76.0-91.5) | 99.8  (98.9-100.0) | NR | NR | ≥3,000 copies/mL |
|  |  |  |  | 83.0  (73.4-90.1) | 100.0  (99.3-100.0) | NR | NR | ≥5,000 copies/mL |
|  |  |  | Venous | 91.4  (84.4-96.0) | 97.2  (95.4-98.5) | NR | NR | ≥1,000 copies/mL |
|  |  |  |  | 90.0  (82.2-95.4) | 99.4  (98.3-99.9) | NR | NR | ≥3,000 copies/mL |
|  |  |  |  | 88.5  (79.9-94.3) | 99.8  (98.9-100.0) | NR | NR | ≥5,000 copies/mL |
| Mwau *et al.* 2021 | HIV | Aptima HIV-1 Quant Dx (Hologic) | Capillary (finger poke) | 92.3  (87.0-95.5) | 92.2  (85.3-96.0) | 94.7  (90.0-97.3) | 88.7  (81.2-93.4) |  |
|  |  |  | Venous | 97.4  (93.6-99.0) | 92.2  (85.3-96.0) | 95.0  (90.4-97.4) | 95.9  (90.0-98.4) |  |
| Rutstein *et al.* 2014 | HIV | m2000SP, m2000RT (Abbott) | Capillary (finger poke) | 100 | 94.9 | 63.2 | 100 | ≥1,000 copies/mL |
|  |  |  |  | 100 | 97.8 | 76.9 | 100 | ≥5,000 copies/mL |
|  |  |  | Venous | 100 | 97.1 | 75.0 | 100 | ≥1,000 copies/mL |
|  |  |  |  | 100 | 98.6 | 83.3 | 100 | ≥5,000 copies/mL |
| Schmitz *et al.* 2017 | HIV | COBAS AmpliPrep/COBAS TaqMan v2.0 (Roche) | Capillary (finger poke) | 88.1  (83.3-92.0) | 94.5  (92.1-96.3) | NR | NR | ≥1,000 copies/mL |
|  |  |  |  | 85.2  (80.0-89.4) | 97.8  (96.2-98.9) | NR | NR | ≥3,000 copies/mL |
|  |  |  |  | 82.2  (76.7-86.9) | 98.8  (97.4-99.6) | NR | NR | ≥5,000 copies/mL |
|  |  |  | Capillary (microtainer) | 90.3  (85.7-93.7) | 94.9  (92.6-96.6) | NR | NR | ≥1,000 copies/mL |
|  |  |  |  | 88.1  (83.3-92.0) | 97.6  (95.9-98.8) | NR | NR | ≥3,000 copies/mL |
|  |  |  |  | 83.9  (78.6-88.3) | 98.6  (97.2-99.4) | NR | NR | ≥5,000 copies/mL |
|  |  |  | Venous | 90.1  (85.7-93.6) | 93.1  (90.6-95.2) | NR | NR | ≥1,000 copies/mL |
|  |  |  |  | 85.2  (80.1-89.4) | 98.0  (96.4-99.1) | NR | NR | ≥3,000 copies/mL |
|  |  |  |  | 83.1  (77.8-87.6) | 98.4  (96.9-99.3) | NR | NR | ≥5,000 copies/mL |
| Tang *et al.* 2017 | HIV | m2000SP, m2000RT (Abbott) | Capillary (finger poke) | 93.0  (88.7-95.2) | 95.0  (92.0-97.4) | NR | NR |  |
|  |  |  |  | 93.0  (85.3-97.1) | 95.0  (90.9-97.6) | NR | NR | Analysis limited to people living with HIV currently receiving ART |
|  |  |  | Venous | 93.0  (89.6-95.8) | 95.0  (91.0-96.8) | NR | NR |  |
|  |  |  |  | 95.0  (87.1-97.9) | 95.0  (90.9-97.6) | NR | NR | Analysis limited to people living with HIV currently receiving ART |
| Tola *et al.* 2021 | HIV | m2000SP, m2000RT (Abbott) | Capillary (finger poke) | 85.6  (81.5–89.6) | 90.9  (88.1–93.8) | NR | NR | ≥1,000 copies/mL |
|  |  |  |  | 71.5  (66.2–76.7) | 96.6  (94.9–98.4 | NR | NR | ≥3,000 copies/mL |
|  |  |  |  | 62.7  (57.1–68.3 | 97.4  (95.9–99.0) | NR | NR | ≥5,000 copies/mL |
|  |  |  | Capillary (microtainer) | 85.2  (81.1-89.3) | 91.5  (88.7-94.3) | NR | NR | ≥1,000 copies/mL |
|  |  |  |  | 72.5  (67.3–77.7) | 96.9  (95.2–98.6) | NR | NR | ≥3,000 copies/mL |
|  |  |  |  | 65.8  (60.3–71.4) | 97.2  (95.5–98.8) | NR | NR | ≥5,000 copies/mL |
|  |  |  | Venous | 89.1  (85.5–92.7) | 86.6  (83.2–90.0) | NR | NR | ≥1,000 copies/mL |
|  |  |  |  | 76.4  (71.5–81.3) | 96.1  (94.2–98.1) | NR | NR | ≥3,000 copies/mL |
|  |  |  |  | 67.2  (61.8–72.7) | 96.6  (95.2–98.6) | NR | NR | ≥5,000 copies/mL |
| Kerschberger *et al.* 2019 | HIV | COBAS AmpliPrep/COBAS TaqMan HIV-1 v2.0 (Roche) | Capillary (finger poke) | 89.2  (79.1-95.6) | 96.8  (94.0-98.5) | NR | NR |  |
|  |  |  | Venous (phlebotomist) | 88.2  (78.1-94.8) | 98.6  (96.5-99.6) | NR | NR |  |
|  |  |  | Venous (laboratory technician) | 85.3  (74.6-92.7) | 94.5  (91.3-96.8) | NR | NR |  |
|  |  | Biocentric | Capillary (finger poke) | 87.1  (76.1-94.3) | 95.4  (92.3-97.5) | NR | NR |  |
|  |  |  | Venous (phlebotomist) | 87.7  (77.2-94.5) | 97.6  (95.2-99.0) | NR | NR |  |
|  |  |  | Venous (laboratory technician) | 89.2  (79.1-95.6) | 94.6  (91.3-96.9) | NR | NR |  |
| Pannus *et al.* 2013 | HIV | NucliSENS Easy Q v2.0 (bioMérieux) | Capillary (finger poke) | 78.6  (59.0-91.7) | 100.0  (98.9-100.0) | 100.0  (84.6-100.0) | 98.2  (96.1-100.0) | ≥1,000 copies/mL |
|  |  |  |  | 69.6  (47.1-86.6) | 100.0  (89.9-100.0) | 100.0  (79.4-100.0) | 97.9  (95.7-99.2) | ≥5,000 copies/mL |
|  |  |  | Venous | 89.3  (71.8-97.7) | 99.7  (98.3-100.0) | 96.2  (80.4-99.9) | 99.1  (97.3-99.8) | ≥1,000 copies/mL |
|  |  |  |  | 60.9  (38.5-80.3) | 100.0  (98.9-100.0) | 100.0  (76.8-100.0) | 97.3  (95.0-98.8) | ≥5,000 copies/mL |
| Biondi *et al.* 2019 | HCV | ARCHITECT Core Antigen Assay (Abbott) | Capillary (finger poke) | 91.8  (84.2-99.5) | NR | NR | NR | ≥3 fmol/L; 1 spot (75 µL) |
|  |  |  |  | 81.6  (70.8-92.5) | NR | NR | NR | ≥10 fmol/L; 1 spot (75 µL) |
|  |  |  |  | 93.9  (87.2-100.0) | NR | NR | NR | ≥3 fmol/L; 2 spot (150 µL) |
|  |  |  |  | 85.7  (75.9-95.5) | NR | NR | NR | ≥10 fmol/L; 2 spot (150 µL) |
|  |  |  | Venous | 91.8  (84.2-99.5) | NR | NR | NR | ≥3 fmol/L; 1 spot (75 µL) |
|  |  |  |  | 87.8  (79.0-97.0) | NR | NR | NR | ≥10 fmol/L; 1 spot (75 µL) |
|  |  |  |  | 93.9  (87.2-100.0) | NR | NR | NR | ≥3 fmol/L; 2 spot (150 µL) |
|  |  |  |  | 87.8  (79.0-97.0) | NR | NR | NR | ≥10 fmol/L; 2 spot (150 µL) |

NPV=negative predictive value; NR=not reported; PPV=positive predictive value; STBBI=sexually transmitted and blood-borne infection;
