# Supplementary material for: Validity of dried blood spot testing for sexually transmitted and blood-borne infections: A narrative systematic review
Source: PLOS Glob Public Health. 2024 Jun 14;4(6):e0003320. doi: 10.1371/journal.pgph.0003320 (PMC11178196; doi:10.1371/journal.pgph.0003320)
Supplement: S4 Table — (DOCX) [file pgph.0003320.s006.docx]

**S4 Table**

*Studies assessing test performance on DBS specimens collected from participants with co-infections*

| Study | STBBI | Index Test | Participants | Sensitivity  (95% CI) | Specificity  (95% CI) | PPV  (95% CI) | NPV  (95% CI) | Notes |
| --- | --- | --- | --- | --- | --- | --- | --- | --- |
| Flores *et al.* 2017 | HCV | Murex AB (DiaSorin) | HIV +ve, HCV –ve | NA | 100.0  (96.3-100.0) | NR | NR |  |
|  |  |  | HIV –ve, HCV +ve | 93.9  (90.0-96.6) | NA | NR | NR |  |
|  |  |  | HIV +ve, HCV –ve | NA | 98.6  (95.1-99.8) | NR | NR |  |
|  |  |  | HIV +ve, HCV +ve | 83.3  (69.7-92.5) | NA | NR | NR |  |
| Flores *et al.* 2021 | HBV | Elecsys HBsAg II (Roche) | Coagulopathy | 100.0 | 100.0 | 100.0 | 100.0 |  |
|  |  |  | Chronic kidney disease | 100.0 | 99.6 | 92.9 | 100.0 |  |
|  |  |  | HIV +ve | 85.0 | 94.7 | 81.0 | 95.9 |  |
|  |  | Elecsys anti-HBc II (Roche) | Coagulopathy | 81.3 | 100.0 | 100.0 | 92.1 |  |
|  |  |  | Chronic kidney disease | 79.6 | 97.1 | 94.7 | 87.8 |  |
|  |  |  | HIV+ve | 97.2 | 88.9 | 85.4 | 98.0 |  |
|  | HCV | Elecsys anti-HCV II (Roche) | Coagulopathy | 83.3 | 96.3 | 95.2 | 86.7 |  |
|  |  |  | Chronic kidney disease | 93.5 | 99.2 | 95.6 | 98.7 |  |
|  |  |  | HIV +ve |  |  |  |  |  |
| Saludes *et al.* 2019 | HCV | In-house RT-qPCR | HIV +ve | 85.4  (72.8-92.8) | NR | NR | NR | ~12 IU/mL |
|  |  |  |  | 87.2  (74.8-94) | NR | NR | NR | ≥12 IU/mL |
|  |  |  |  | 97.6  (87.7-99.6) | NR | NR | NR | ≥1,000 IU/mL |
|  |  |  |  | 97.6  (87.7-99.6) | NR | NR | NR | ≥3,000 IU/mL |
|  |  |  |  | 100.0  (91.0-100.0) | NR | NR | NR | ≥50,000 IU/mL |
|  |  |  | HIV -ve | 89.2  (83.2-93.2) | NR | NR | NR | ~12 IU/mL |
|  |  |  |  | 91.0  (85.2-94.7) | NR | NR | NR | ≥12 IU/mL |
|  |  |  |  | 95.6  (90.7-98.0) | NR | NR | NR | ≥1,000 IU/mL |
|  |  |  |  | 97.0  (92.6-98.8) | NR | NR | NR | ≥3,000 IU/mL |
|  |  |  |  | 100.0  (96.9-100.0) | NR | NR | NR | ≥50,000 IU/mL |

NPV=negative predictive value; NA = not applicable; NR=not reported; PPV=positive predictive value; STBBI=sexually transmitted and blood-borne infection;
