# Supplementary material for: Validity of dried blood spot testing for sexually transmitted and blood-borne infections: A narrative systematic review
Source: PLOS Glob Public Health. 2024 Jun 14;4(6):e0003320. doi: 10.1371/journal.pgph.0003320 (PMC11178196; doi:10.1371/journal.pgph.0003320)
Supplement: S5 Table — (DOCX) [file pgph.0003320.s007.docx]

**S5 Table**

*Studies assessing test performance on DBS specimens collected from participants undergoing ART and ART-naïve participants*

| Study | STBBI | Index Test | Participants | Sensitivity  (95% CI) | Specificity  (95% CI) | PPV  (95% CI) | NPV  (95% CI) | Notes |
| --- | --- | --- | --- | --- | --- | --- | --- | --- |
| Alvarez *et al.* 2015 | HIV | COBAS AmpliPrep/COBAS TaqMan HIV-1 Quantitative Test v2.0 (Roche) | ART-naïve | 94.7  (74.0-99.9) | 90.5  (77.4-97.3) | NR | NR |  |
|  |  |  | Undergoing ART | 96.2  (86.8-99.5) | 90.5  (80.4-96.4) | NR | NR |  |
|  |  |  | All | 95.8  (88.1-99.1) | 89.5  (82.0-94.7) | NR | NR |  |
|  |  | Versant HIV-1 RNA 1.0 (Siemens) | ART-naïve | 84.2  (60.4-96.6) | 100.0  (91.0-100.0) | NR | NR |  |
|  |  |  | Undergoing ART | 64.7  (50.4-77.6) | 100.0  (93.4-100.0) | NR | NR |  |
|  |  |  | All | 70.0  (57.9-80.4) | 100.0  (96.1-100.0) | NR | NR |  |
| Balinda *et al.* 2016 | HIV | In-house RT-qPCR | ART-naïve | 79.4 | 54.5 | 89.0 | 36.4 |  |
|  |  |  | Undergoing ART (>6 months) | 75.7 | 95.5 | 65.1 | 97.3 |  |
|  |  |  | Undergoing ART (12-36 months) | 88.9 | 98.1 | 72.7 | 99.3 |  |
| Ondoa *et al.* 2014 |  | COBAS AmpliPrep/COBAS TaqMan HIV-1 Quantitative Test v2.0 (Roche) | ART-naïve | 61.9 | 99.0 | NR | NR | ≥5,000 copies/mL  Principal outcome was virological failure. Inadequate storage conditions and/or the lack of experience of the operator may have contributed to poor sensitivity. |
|  |  |  | Undergoing ART | 9.0 | 100.0 | NR | NR | ≥5,000 copies/mL |
| Taieb *et al.* 2016 |  | m2000SP, m2000RT (Abbott) | Undergoing ART (<6 months) | 87.5  (47.3-99.7) | 87.1  (70.1-96.4) | NR | NR |  |
|  |  |  | Undergoing ART (≥6 months) | 85.2  (66.3-95.6) | 99.0  (94.3-100.0) | NR | NR |  |
|  |  |  | All | 90.1  (80.7-95.9) | 96.2  (91.4-98.8) | NR | NR |  |
